# *In vitro* phosphorylation of BRCA2 by the checkpoint kinase CHEK2

**DOI:** 10.1038/sj.bjc.6604644

**Published:** 2008-09-16

**Authors:** S Kim, G Mohapatra, D A Haber

**Affiliations:** 1 Massachusetts General Hospital Cancer Center, Harvard Medical School, Massachusetts General Hospital, Charlestown, MA 02129, USA; 2 Department of Pathology, Harvard Medical School, Massachusetts General Hospital, Charlestown, MA 02129, USA

**Keywords:** BRCA1 or 2 (breast cancer gene 1 or 2), CHK2 or CHEK2 (checkpoint kinase 2), IR (ionising radiation), GTG banding (G-bands after trypsin and giemsa), NLS (nuclear localisation signal)

## Abstract

Germline mutations in both *BRCA2* and *CHEK2* are associated with an increased risk for male breast cancer. To search for potential interactions between the products of these breast cancer susceptibility genes, we undertook systematic mapping of BRCA2 for potential phosphorylation sites by CHEK2. *In vitro* kinase assays and mass spectrometric analysis identified a 50 amino-acid fragment within the N-terminus of BRCA2 potentially targeted by CHEK2, containing two major phosphopeptides. Inducible overexpression of this peptide, but not a derivative with mutated phosphorylation sites, leads to increased chromosome fragmentation and suppression of cellular proliferation. These results suggest a link between CHEK2 and BRCA2 pathways, which may contribute to the spectrum of cancers associated with germline CHEK2 mutations.

CHEK2 (also known as CHK2) encodes an ATM-dependent serine-threonine kinase that modulates a conserved DNA damage checkpoint pathway (Ahn *et al*, 2004). Although its orthologs Rad53 (*Saccharomyces cerevisiae*) and Cds1 (*Schizosaccharomyces pombe)* are integral components of the G2/M DNA damage checkpoint (Sanchez *et al*, 1996; Rhind *et al*, 2000), mammalian CHEK2 has been reported to mediate phosphorylation of a number of targets, implicated in multiple aspects of cell cycle progression and DNA damage repair (Zhou and Elledge, 2000; Bartek *et al*, 2001). By analogy with its yeast orthologs, CHEK2 was first shown to phosphorylate Cdc25C on Ser216, leading to its cytoplasmic sequestration by 14-3-3 proteins, and preventing the dephosphorylation and activation of Cyclin B/Cdc2, thus mediating a G2 arrest (Peng *et al*, 1997; Sanchez *et al*, 1997; Matsuoka *et al*, 1998). Subsequently, CHEK2 was shown to have additional targets, including Cdc25A (Ser123) leading to its destabilisation and activating the S-phase checkpoint (Mailand *et al*, 2000; Falck *et al*, 2001); p53 (Ser20), linked to its activation following ionising radiation (IR) and modulating a G1/S arrest (Chehab *et al*, 2000; Hirao *et al*, 2000; Shieh *et al*, 2000); and BRCA1 (Ser988), implicated in its altered subnuclear localisation (Lee *et al*, 2000). Although these pathways have been studied primarily in cancer cell lines, a number of mouse models of CHEK2 inactivation have provided some insight into the physiologically relevant pathways (Hirao *et al*, 2002; Takai *et al*, 2002). These have demonstrated a mild attenuation of p53 function, with modest tumorigenesis, but significant synergistic effects following inactivation of both CHEK2 and Brca1 (McPherson *et al*, 2004).

In humans, CHEK2 inactivation is thought to mediate a moderately increased risk for breast and possibly prostate cancers (Allinen *et al*, 2001; Meijers-Heijboer *et al*, 2002; Seppala *et al*, 2003; Cybulski *et al*, 2004). Germline mutations were initially identified in families with Li Fraumeni-like multicancer syndromes lacking the characteristic mutation in p53 (Bell *et al*, 1999). Subsequent population-based studies have shown that a recurrent loss of function CHEK2 mutant, CHEK21100delC is present in approximately 1% of the population and in 5% of familial breast cancer families lacking a BRCA1 or BRCA2 mutation (Meijers-Heijboer *et al*, 2002; Fernandez-Novoa *et al*, 2004). The fact that BRCA1/2 kindreds displayed no increase in CHEK21100delC mutation frequency was taken as evidence that CHEK2 may function in a similar pathway, thus obviating the need for redundant mutations. Most significantly, the frequency of CHEK21100delC in breast cancer families with a case of male breast cancer was as high as 13% (Meijers-Heijboer *et al*, 2002). Given the relatively unique nature of the link between BRCA2 mutations and male breast cancer, we therefore sought evidence of a functional interaction between BRCA2 and CHEK2.

## Materials and methods

### Generation of constructs and conditionally expressing cell lines

Overlapping serial fragments of BRCA2, with the exception of the extreme C terminus, which could not be expressed *in vitro*, were constructed by PCR and introduced into pGEX2T to express GST-fusion proteins. Proteins were expressed in BL21 cells, followed by Glutathione Sepharose 4B bead purification, and dialysis (20 mM HEPES, pH 7.4, 20% glycerol, and 0.5 mM PMSF) of purified proteins. The B2P fragment, containing either wild-type sequence of 50 amino-acids spanning the CHEK2 targets, or a mutated version was fused to a Flag at the N-terminus and SV40 NLS at the C-terminus and cloned into the tetracycline-regulated vector pUHD10.3. Inducible expression of a wild-type B2P (WT), a mutant version with all four serine/threonine to alanine/valine, or a previously described BRC repeat fragment, BRC4 (Chen *et al*, 1999) was achieved by co-transfecting a U2OS founder cell line with the corresponding pUHD10.3 construct, along with a plasmid encoding hygromycin resistance. Hygromycin-resistant clones were selected for comparable levels of inducible B2P expression by western blot analysis, and at least two independent clones were selected for each construct for functional assays.

### *In vitro* kinase assays

*In vitro* kinase assays were performed using either cellular CHEK2 derived from U2OS cells with inducible expression, immunoprecipitated using anti-Flag antibody, 1 h after *γ*-irradiation (10 Gy), or purified bacterially expressed GST-CHEK2 proteins. Various fragments of GST-BRCA2 (B2P-WT or -mutants) were used as substrates in a 30-min incubation using 20 mM HEPES (pH 7.4), 10 mM MgCl_2_, 10 mM MnCl_2_, 40 *μ*M ATP, and 15 *μ*Ci [*γ*-^32^P]ATP at 30°C.

### Cell growth assays and chromosome spreading and GTG banding

Equal numbers of cells (5 × 10^4^) with inducible expression of B2P-WT, B2P-M9, or BRC4 were plated onto six-well plates in the presence of tetracycline (1 *μ*g ml^−1^). Tetracycline was withdrawn after 24 h and viable cells were counted at the indicated times. For chromosome analysis, equal numbers of cells (5 × 10^5^) were plated, followed by inducible expression of the appropriate constructs and treatment of cells with colchicine (10 *μ*g ml^−1^) for 20 h. Cells were then treated with trypsin and Giemsa for GTG banding as described (Thalhammer *et al*, 2001). Individual metaphases were photographed and scored blindly for abnormal structures and broken chromosomes.

### Statistical methods

The comparison of mean fragment/metaphase between two groups was performed by two-sided Student's *t*-test. We performed two comparisons (i.e., comparisons of WT *vs* BRC4, and M9 *vs* BRC4), and the Bonferroni's correction was applied to the significance level of each comparison. A *P*-value <0.025 was considered as a statistically significant result.

## Results and discussion

**Figure 1:**
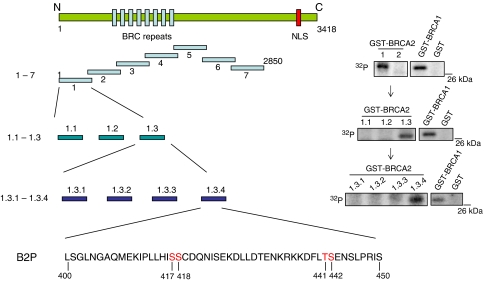
Phosphorylation of the N-terminal fragment of BRCA2 (B2P) by CHEK2 *in vitro*. Schematic representation of BRCA2 in seven overlapping GST-fusion proteins, designated 1–7, and used for *in vitro* kinase assays. Fragment 1 was further subdivided into the three overlapping GST-fusion proteins, 1.1, 1.2, and 1.3, and the fragment 1.3 region was divided again into fragments 1.3.1, 1.3.2, 1.3.3, and 1.3.4. The final peptide containing specific CHEK2 phosphorylation sites was designated B2P: it has 50 amino acids with four putative substrate sites (shown in red), identified by mass-spectrometric analysis. The mass-spectrometric analysis was performed by using fragments of BRCA2 expressed from bacteria as GST-fusion proteins with either bacterially expressed GST-CHEK2 or immunoprecipitated Flag-CHEK2 from IR-treated tissue culture cells. The right panel shows the *in vitro* CHEK2 phosphorylation assay for each step of analysis with BRCA2 fragments. GST-BRCA1 fragment containing S988 and GST alone were used as positive and negative controls.

**Figure 2:**
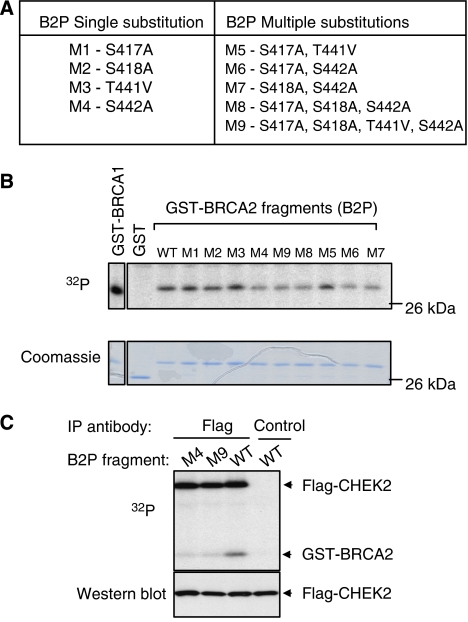
BRCA2 residues targeted by CHEK2 phosphorylation. (**A**) The four residues containing putative phosphorylation sites in the BRCA2 fragment B2P were mutagenised individually or in combination as shown. (**B**) Phosphorylation of wild type (WT) or mutant forms of B2P fragments by CHEK2. GST-fusion proteins were incubated with bacterially expressed GST-CHEK2 proteins in the presence of [*γ*-^32^P]ATP. This *in vitro* kinase assay was repeated three times with similar results. (**C**) Phosphorylation of B2P by immunoprecipitated CHEK2, following *in vivo* activation by ionising radiation. Flag-tagged CHEK2 protein was induced for 24 h in U2OS cells with tetracycline-regulated expression, and lysates were collected 1 h following treatment with 10 Gy and immunoprecipitated using either anti-Flag or nonspecific (control) antibodies. Wild-type (WT) or mutant (M4, M9) GST-fusion B2P fragments were incubated with the immunoprecipitates in the presence of ^32^P. Phosphorylation of wild-type, but not mutant, GST-BRCA2 fragments is evident in CHEK2 immunoprecipitates. Flag-CHEK2 itself shows autophosphorylation as well. Total lysates (10 *μ*g) were immunoblotted to show comparable expression of Flag-CHEK2 (western blot control).

To search for potential CHEK2 phosphorylation sites within BRCA2, we first generated a series of overlapping glutathione *S*-transferase (GST)-fusion proteins spanning the BRCA2 coding sequence, with the exception of the extreme C terminus, which could not be expressed *in vitro* (Figure 1). Purified GST-BRCA2 fragments were incubated with either bacterially expressed GST-CHEK2 or with immunoprecipitates of CHEK2 derived from X-irradiated (10 Gy) U2OS cells expressing a tetracycline-regulated Flag-tagged construct. A single N-terminal fragment of BRCA2 (amino acids 1–450) showed reproducible *in vitro* phosphorylation by both GST-CHEK2 and immunoprecipitated CHEK2 proteins, comparable in intensity to the ^988^Serine-containing BRCA1 fragment previously identified as a CHEK2 target (Figure 1, data not shown). Through a series of further subcloning experiments, the putative CHEK2 target sequence was narrowed to 50 amino-acid residues (aa 400–450), referred to as B2P. (Figures 1 and 2A). Mass-spectrometric analysis of B2P, following coincubation with either GST-CHEK2 or immunoprecipitated CHEK2 from irradiated cells, identified four phosphorylated residues: ^417^Serine, ^418^Serine, ^442^Serine, and ^441^Threonine (Figures 1 and 2B, C) and ^418^Serine is well conserved in most mammals (Supplementary data).

To test the contribution of each Serine and Threonine residues to CHEK2-mediated phosphorylation, we undertook site-directed mutagenesis of B2P, substituting unphosphorylatable residues (Serine to Alanine, and Threonine into Valine), either alone or in combination (mutant peptides M1-9, Figure 2B). Fragments containing single, double, triple, or quadruple mutations were used as substrates for *in vitro* GST-CHEK2-mediated phosphorylation. Of peptides with a single substitution, only M4 (targeting ^442^Ser) showed a reduction in CHEK2-dependent phosphorylation using either GST-CHEK2 or immunoprecipitated CHEK2 from irradiated cells (Figure 2B and C). Peptides with multiple substitutions including S442A (M6, M7, M8, M9) also showed reduced CHEK2 phosphorylation, whereas a multisubstituted peptide with an unaltered ^442^Ser (M5) had preserved CHEK2 phosphorylation (Figure 2B). Taken together, these studies point to ^442^Ser as the potentially major CHEK2 substrate in BRCA2, with additional minor targets including ^417^Serine, ^418^Serine, and ^441^Threonine.

**Figure 3:**
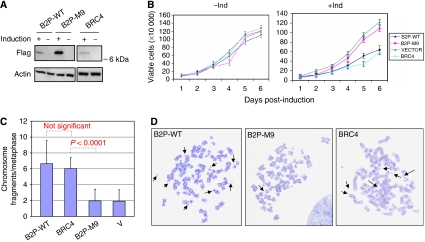
Suppression of cellular proliferation and induction of chromosome fragmentation by overexpression of wild-type B2P. (**A**) Inducible expression of B2P. Immunoblotting (*α*-Flag) analysis of cells with inducible expression of the wild-type Flag-B2P-NLS (B2P-WT), mutant form (B2P-M9), or the known BRCA2 inhibitory peptide BRC4 (actin loading control). (**B**) Inhibition of cell proliferation by expression of B2P. Cells with inducible expression of B2P, mutant (B2P-M9), BRC4 or vector were grown in the presence (−Ind) or absence (+Ind) of tetracycline. Viable cells were counted at the indicated times. Experiments were performed in triplicate with standard deviation shown. (**C**) Increased chromosome fragmentation following induction of B2P. The mean number of chromosome fragments per metaphase spread (*N*=50) is shown for cells with inducible expression of B2P-WT, mutant (B2P-M9), BRC4 or vector alone. The mean fragment/metaphase levels between B2P-M9 and BRC4 were significantly different (*P*<0.0001). (**D**) Representative metaphase spread from cells expressing either B2P-WT, B2P-M9, or BRC4. Arrowheads indicate fragmented chromosomes.

Robust and specific assays of BRCA2 function are limited, but *brca2*-deficient cells are known to have a striking increase in chromosomal defects (Patel *et al*, 1998; Venkitaraman, 2002). To define a potential functional assay for BRCA2 phosphorylation at residues localised to the B2P fragment, we generated cell lines with tetracycline-regulated inducible expression of this peptide or a mutant peptide with all four substituted CHEK2-target residues. The osteosarcoma cell line U2OS was selected for these experiments, given its near diploid and stable karyotype, wild-type endogenous p53, and the availability of tightly regulated tetracycline-regulatable founder cell lines. Cell lines were generated, with inducible expression of wild-type or mutated (M9) B2P peptide, linked to a nuclear localisation signal (NLS) and a Flag epitope. In multiple cell lines, tightly regulated expression was achievable with both constructs, although the mutant was consistently induced to higher levels than the wild-type peptide (Figure 3A). As control, we also generated cells with inducible expression of BRC4 (Figure 3A), a BRCT-containing fragment of BRCA2, which has been reported to disrupt the RAD51-dependent function of BRCA2 (Chen *et al*, 1999). Inducible expression of wild-type B2P, but not the M9 mutant, led to a reduction in cell proliferation, similar to that observed following induction of BRC4 (Figure 3B). As the inactivation of BRCA2 is known to result in spontaneous disruption of chromosome structure (Chen *et al*, 1999), we examined chromosome spreads of U2OS cells with inducible expression of B2P using GTG banding. A reproducible increase in chromosome fragments per metaphase was observed 48 h following induction of wild-type B2P, but not the M9 mutant (Figure 3C and D). The effect of B2P peptide expression was comparable to that of BRC4 (Figure 3C and D). There was no statistically significant difference in mean fragment/metaphase between B2P-WT and BRC4. The mean fragment/metaphase levels between B2P-M9 and BRC4 were significantly different (*P*<0.0001). Thus, ectopic overexpression of the BRCA2 fragment containing intact CHEK2 phosphorylation sites may disrupt endogenous gene product function, leading to chromosomal instability.

## Concluding remarks

Our studies suggest a potential functional link between the CHEK2 kinase and the BRCA2 tumour suppressor, which may explain in part the increased incidence of male breast cancer observed in families with germline mutations in both of these genes (Couch *et al*, 1996; Haraldsson *et al*, 1998; Meijers-Heijboer *et al*, 2002). While factors affecting BRCA2 protein function are poorly understood, two other kinases have recently been implicated in its regulation (Lin *et al*, 2003; Lee *et al*, 2004; Esashi *et al*, 2005). CDK-dependent phosphorylation of the C-terminal BRCT domain (Ser 3291) has been shown to modulate the BRCA2–RAD51 interaction in a cell cycle-dependent manner. This interaction was disrupted by ectopic expression of BRCA2-derived peptides containing the phosphorylation target site (Esashi *et al*, 2005). In addition, polo-like kinase 1 (PLK1) has been shown to phosphorylate the N terminus of BRCA2, modulating its dissociation from a transcriptional co-activator protein, P/CAF, with intrinsic histone acetyltransferase activity (Lin *et al*, 2003; Lee *et al*, 2004). The effect of CHEK2 on BRCA2 remains to be confirmed *in vivo*, as does its effect on potential functional properties of BRCA2. Nonetheless, the potential modulation by CHEK2 of both BRCA1 and BRCA2 would point to an important function for this checkpoint kinase in the DNA damage repair pathways implicated in the development of breast cancer.

## References

[bib1] Ahn J, Urist M, Prives C (2004) The CHK2 protein kinase. DNA Repair (Amst) 3: 1039–1047 15279791 10.1016/j.dnarep.2004.03.033

[bib2] Allinen M, Huusko P, Mantyniemi S, Launonen V, Winqvist R (2001) Mutation analysis of the CHK2 gene in families with hereditary breast cancer. Br J Cancer 85: 209–212 11461078 10.1054/bjoc.2001.1858 PMC2364033

[bib3] Bartek J, Falck J, Lukas J (2001) CHK2 kinase–a busy messenger. Nat Rev Mol Cell Biol 2: 877–886 11733767 10.1038/35103059

[bib4] Bell DW, Varley JM, Szydlo TE, Kang DH, Wahrer DC, Shannon KE, Lubratovich M, Verselis SJ, Isselbacher KJ, Fraumeni JF, Birch JM, Li FP, Garber JE, Haber DA (1999) Heterozygous germ line hCHK2 mutations in Li-Fraumeni syndrome. Science 286: 2528–2531 10617473 10.1126/science.286.5449.2528

[bib5] Chehab NH, Malikzay A, Appel M, Halazonetis TD (2000) CHK2/hCds1 functions as a DNA damage checkpoint in G(1) by stabilizing p53. Genes Dev 14: 278–288 10673500 PMC316357

[bib6] Chen CF, Chen PL, Zhong Q, Sharp ZD, Lee WH (1999) Expression of BRC repeats in breast cancer cells disrupts the BRCA2-Rad51 complex and leads to radiation hypersensitivity and loss of G(2)/M checkpoint control. J Biol Chem 274: 32931–32935 10551859 10.1074/jbc.274.46.32931

[bib7] Couch FJ, Farid LM, DeShano ML, Tavtigian SV, Calzone K, Campeau L, Peng Y, Bogden B, Chen Q, Neuhausen S, Shattuck-Eidens D, Godwin AK, Daly M, Radford DM, Sedlacek S, Rommens J, Simard J, Garber J, Merajver S, Weber BL (1996) BRCA2 germline mutations in male breast cancer cases and breast cancer families. Nat Genet 13: 123–125 8673091 10.1038/ng0596-123

[bib8] Cybulski C, Huzarski T, Gorski B, Masojc B, Mierzejewski M, Debniak T, Gliniewicz B, Matyjasik J, Zlowocka E, Kurzawski G, Sikorski A, Posmyk M, Szwiec M, Czajka R, Narod SA, Lubinski J (2004) A novel founder CHEK2 mutation is associated with increased prostate cancer risk. Cancer Res 64: 2677–2679 15087378 10.1158/0008-5472.can-04-0341

[bib9] Esashi F, Christ N, Gannon J, Liu Y, Hunt T, Jasin M, West SC (2005) CDK-dependent phosphorylation of BRCA2 as a regulatory mechanism for recombinational repair. Nature 434: 598–604 15800615 10.1038/nature03404

[bib10] Falck J, Mailand N, Syljuasen RG, Bartek J, Lukas J (2001) The ATM-CHK2-Cdc25A checkpoint pathway guards against radioresistant DNA synthesis. Nature 410: 842–847 11298456 10.1038/35071124

[bib11] Fernandez-Novoa MC, Vargas MT, Granell MR, Carreto P (2004) Prenatal diagnosis of *de novo* trisomy 1(q21-qter)der(Y)t(Y;1) in a malformed live born. Prenat Diagn 24: 414–417 15229838 10.1002/pd.847

[bib12] Haraldsson K, Loman N, Zhang QX, Johannsson O, Olsson H, Borg A (1998) BRCA2 germ-line mutations are frequent in male breast cancer patients without a family history of the disease. Cancer Res 58: 1367–1371 9537231

[bib13] Hirao A, Cheung A, Duncan G, Girard PM, Elia AJ, Wakeham A, Okada H, Sarkissian T, Wong JA, Sakai T, De Stanchina E, Bristow RG, Suda T, Lowe SW, Jeggo PA, Elledge SJ, Mak TW (2002) CHK2 is a tumor suppressor that regulates apoptosis in both an ataxia telangiectasia mutated (ATM)-dependent and an ATM-independent manner. Mol Cell Biol 22: 6521–6532 12192050 10.1128/MCB.22.18.6521-6532.2002 PMC135625

[bib14] Hirao A, Kong YY, Matsuoka S, Wakeham A, Ruland J, Yoshida H, Liu D, Elledge SJ, Mak TW (2000) DNA damage-induced activation of p53 by the checkpoint kinase CHK2. Science 287: 1824–1827 10710310 10.1126/science.287.5459.1824

[bib15] Lee JS, Collins KM, Brown AL, Lee CH, Chung JH (2000) hCds1-mediated phosphorylation of BRCA1 regulates the DNA damage response. Nature 404: 201–204 10724175 10.1038/35004614

[bib16] Lee M, Daniels MJ, Venkitaraman AR (2004) Phosphorylation of BRCA2 by the Polo-like kinase Plk1 is regulated by DNA damage and mitotic progression. Oncogene 23: 865–872 14647413 10.1038/sj.onc.1207223

[bib17] Lin HR, Ting NS, Qin J, Lee WH (2003) M phase-specific phosphorylation of BRCA2 by Polo-like kinase 1 correlates with the dissociation of the BRCA2-P/CAF complex. J Biol Chem 278: 35979–35987 12815053 10.1074/jbc.M210659200

[bib18] Mailand N, Falck J, Lukas C, Syljuasen RG, Welcker M, Bartek J, Lukas J (2000) Rapid destruction of human Cdc25A in response to DNA damage. Science 288: 1425–1429 10827953 10.1126/science.288.5470.1425

[bib19] Matsuoka S, Huang M, Elledge SJ (1998) Linkage of ATM to cell cycle regulation by the CHK2 protein kinase. Science 282: 1893–1897 9836640 10.1126/science.282.5395.1893

[bib20] McPherson JP, Lemmers B, Hirao A, Hakem A, Abraham J, Migon E, Matysiak-Zablocki E, Tamblyn L, Sanchez-Sweatman O, Khokha R, Squire J, Hande MP, Mak TW, Hakem R (2004) Collaboration of Brca1 and CHK2 in tumorigenesis. Genes Dev 18: 1144–1153 15131084 10.1101/gad.1192704 PMC415639

[bib21] Meijers-Heijboer H, van den Ouweland A, Klijn J, Wasielewski M, de Snoo A, Oldenburg R, Hollestelle A, Houben M, Crepin E, van Veghel-Plandsoen M, Elstrodt F, van Duijn C, Bartels C, Meijers C, Schutte M, McGuffog L, Thompson D, Easton D, Sodha N, Seal S, Barfoot R, Mangion J, Chang-Claude J, Eccles D, Eeles R, Evans DG, Houlston R, Murday V, Narod S, Peretz T, Peto J, Phelan C, Zhang HX, Szabo C, Devilee P, Goldgar D, Futreal PA, Nathanson KL, Weber B, Rahman N, Stratton MR (2002) Low-penetrance susceptibility to breast cancer due to CHEK2(^*^)1100delC in noncarriers of BRCA1 or BRCA2 mutations. Nat Genet 31: 55–59 11967536 10.1038/ng879

[bib22] Patel KJ, Yu VP, Lee H, Corcoran A, Thistlethwaite FC, Evans MJ, Colledge WH, Friedman LS, Ponder BA, Venkitaraman AR (1998) Involvement of Brca2 in DNA repair. Mol Cell 1: 347–357 9660919 10.1016/s1097-2765(00)80035-0

[bib23] Peng CY, Graves PR, Thoma RS, Wu Z, Shaw AS, Piwnica-Worms H (1997) Mitotic and G2 checkpoint control: regulation of 14-3-3 protein binding by phosphorylation of Cdc25C on serine-216. Science 277: 1501–1505 9278512 10.1126/science.277.5331.1501

[bib24] Rhind N, Baber-Furnari BA, Lopez-Girona A, Boddy MN, Brondello JM, Moser B, Shanahan P, Blasina A, McGowan C, Russell P (2000) DNA damage checkpoint control of mitosis in fission yeast. Cold Spring Harb Symp Quant Biol 65: 353–359 12760050 10.1101/sqb.2000.65.353

[bib25] Sanchez Y, Desany BA, Jones WJ, Liu Q, Wang B, Elledge SJ (1996) Regulation of RAD53 by the ATM-like kinases MEC1 and TEL1 in yeast cell cycle checkpoint pathways. Science 271: 357–360 8553072 10.1126/science.271.5247.357

[bib26] Sanchez Y, Wong C, Thoma RS, Richman R, Wu Z, Piwnica-Worms H, Elledge SJ (1997) Conservation of the Chk1 checkpoint pathway in mammals: linkage of DNA damage to Cdk regulation through Cdc25. Science 277: 1497–1501 9278511 10.1126/science.277.5331.1497

[bib27] Seppala EH, Ikonen T, Mononen N, Autio V, Rokman A, Matikainen MP, Tammela TL, Schleutker J (2003) CHK2 variants associate with hereditary prostate cancer. Br J Cancer 89: 1966–1970 14612911 10.1038/sj.bjc.6601425 PMC2394451

[bib28] Shieh SY, Ahn J, Tamai K, Taya Y, Prives C (2000) The human homologs of checkpoint kinases Chk1 and Cds1 (CHK2) phosphorylate p53 at multiple DNA damage-inducible sites. Genes Dev 14: 289–300 10673501 PMC316358

[bib29] Takai H, Naka K, Okada Y, Watanabe M, Harada N, Saito S, Anderson CW, Appella E, Nakanishi M, Suzuki H, Nagashima K, Sawa H, Ikeda K, Motoyama N (2002) CHK2-deficient mice exhibit radioresistance and defective p53-mediated transcription. EMBO J 21: 5195–5205 12356735 10.1093/emboj/cdf506 PMC129029

[bib30] Thalhammer S, Koehler U, Stark RW, Heckl WM (2001) GTG banding pattern on human metaphase chromosomes revealed by high resolution atomic-force microscopy. J Microsc 202: 464–467 11422667 10.1046/j.1365-2818.2001.00909.x

[bib31] Venkitaraman AR (2002) Cancer susceptibility and the functions of BRCA1 and BRCA2. Cell 108: 171–182 11832208 10.1016/s0092-8674(02)00615-3

[bib32] Zhou BB, Elledge SJ (2000) The DNA damage response: putting checkpoints in perspective. Nature 408: 433–439 11100718 10.1038/35044005

