# Supplementary material for: In vitro phosphorylation of BRCA2 by the checkpoint kinase CHEK2
Source: Br J Cancer. 2008 Sep 16;99(8):1302–6. doi: 10.1038/sj.bjc.6604644 (PMC2570522; doi:10.1038/sj.bjc.6604644)
Supplement: Supplementary data [file 6604644x1.ppt]

## Slide 1
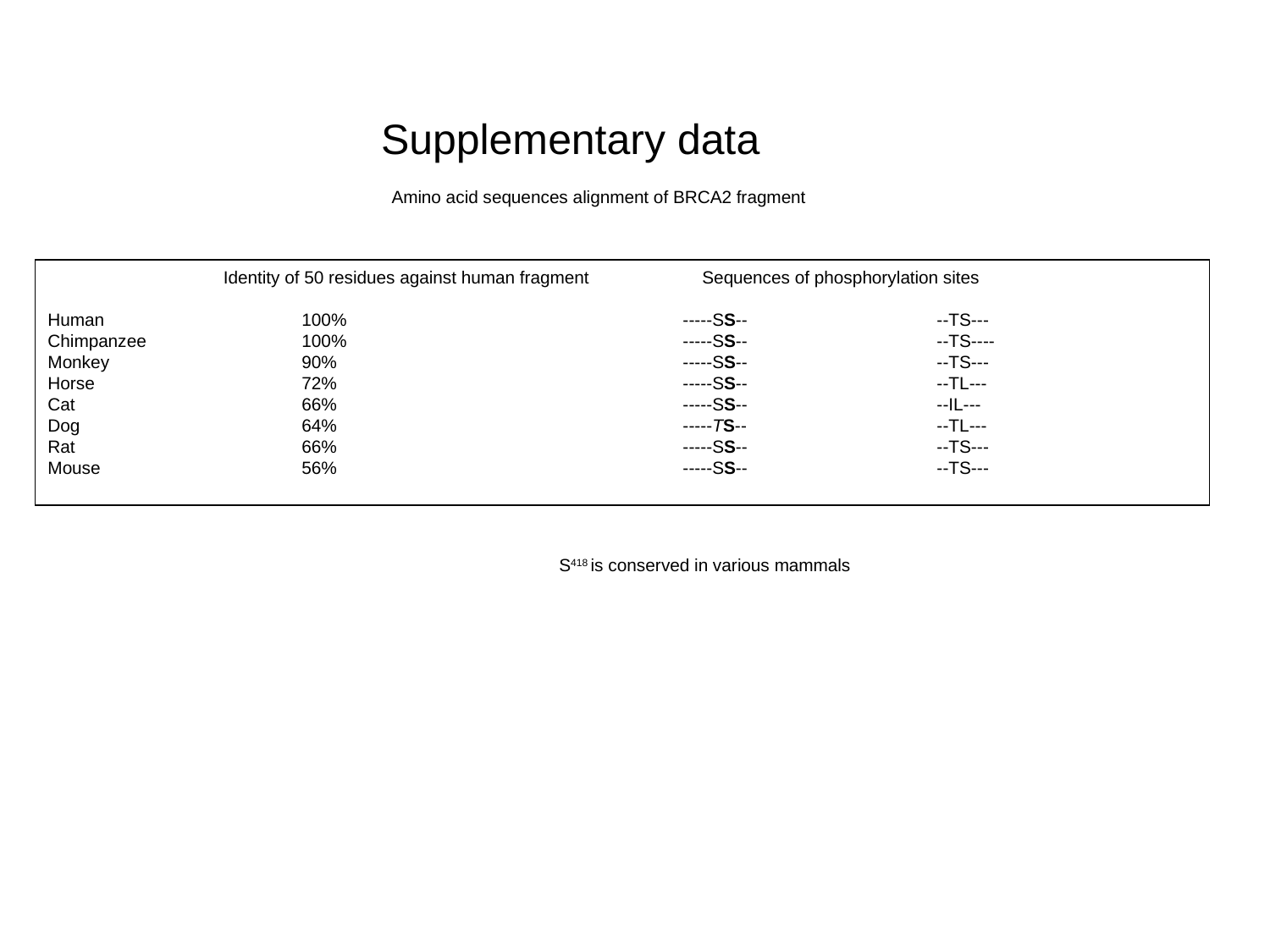

Supplementary data
Amino acid sequences alignment of BRCA2 fragment
 	 Identity of 50 residues against human fragment	 Sequences of phosphorylation sites
Human		100%			-----SS--		--TS---
Chimpanzee 		100%			-----SS--		--TS----
Monkey 		90%			-----SS--		--TS---
Horse		72%			-----SS--		--TL---
Cat		66%			-----SS--		--IL---
Dog		64%			-----TS--		--TL---
Rat		66%			-----SS--		--TS---
Mouse		56%			-----SS--		--TS---
S418 is conserved in various mammals
